# Assessment of high spatial resolution satellite imagery for monitoring riparian vegetation: riverine management in the smallholding

**DOI:** 10.1007/s10661-022-10667-8

**Published:** 2022-11-07

**Authors:** Paula Rivas-Fandiño, Carolina Acuña-Alonso, Ana Novo, Fernando António Leal Pacheco, Xana Álvarez

**Affiliations:** 1 Agroforestry Group, School of Forestry Engineering, University of Vigo, 36005 Pontevedra, Spain; 2 Geotech Group, Department of Natural Resources and Environmental Engineering, School of Mining Engineering, CINTECX, University of Vigo, 36310 Vigo, Spain; 3 Center of Chemistry of Vila Real, University of Trás-Os-Montes e Alto Douro, Ap. 1013, 5001-801 Vila Real, Portugal

**Keywords:** WorldView-2, QBR index, RSQI index, River ecosystem

## Abstract

Riverine habitats are essential ecotones that bridge aquatic and terrestrial ecosystems, providing multiple ecosystem services. This study analyses the potential use of high-resolution satellite imagery, provided by the WorldView-2 satellite, in order to assess its viability for monitoring riparian ecosystems. It is performed by calculating the riparian strip quality index (RSQI) and calibrating it with the riparian quality index (QBR). The methodology was implemented in the Umia River, which is characterised by elevated anthropogenic pressures (located in the northwest of Spain). The results obtained by the method have a 92% of veracity and a kappa coefficient of 0.88. The average quality value obtained for the RSQI index was 71.57, while the average value for the QBR was 55.88. This difference could be attributed to the fact that the former does not differ between autochthonous and non-autochthonous vegetation. The areas with more accurate mapping corresponded to stretches of vegetation with optimal cover (80–50%), with good connectivity with the adjacent forest ecosystem and few or no presence of invasive plants. The worst-scoring sites had the next characteristics: low connectivity (< 10%), low forest cover (< 10%) and a higher presence of invasive plants. The degradation of vegetation could be explained by the presence of agriculture and deficient land use rationing caused by the type of ownership of the study area. The application of this index through satellite images will facilitate the environmental governance of multiple ecosystems and in special riparian ecosystems, obtaining a quick and objective methodology, easily replicable in other basins.

**Graphical Abstract:**

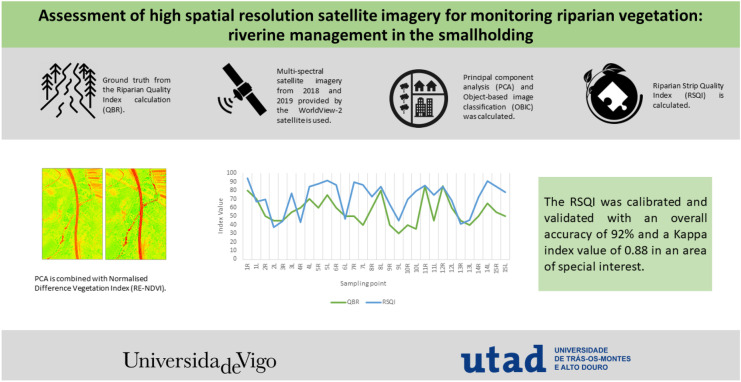

**Supplementary Information:**

The online version contains supplementary material available at 10.1007/s10661-022-10667-8.

## Introduction

Land use changes have ecological consequences for the health of terrestrial and aquatic ecosystems (Bruno et al., 2014). Deforestation, as well as e.g. replacement of native species by allochthonous species (White & Greer, 2006), or the expansion of agricultural and livestock lands, is an upward trend. One of the main implicit consequences of land use change is the revision of ecological integrity (Clerici et al., 2014). It affects the conservation and support of the necessary processes that act in the self-organisation of ecological systems (Barkmann et al., 2001). All this causes a process of degradation, substitution, and elimination, which is even more noticeable in the lands near rivers, whose banks are fertile lands due to the proximity to the watercourses, causing a high demand for exploitation. As a consequence, riverine vegetation is high because of multiple impacts that disturb it (Zermeño‐Hernández et al., 2020).

Riparian zones are transitional areas between fluvial and terrestrial ecosystems that provide heterogeneous microhabitats (Rykken et al., 2007) for terrestrial and aquatic populations, thus supporting biodiverse communities (Ramey & Richardson, 2017). The riverine zone is mainly characterised by a high spatial and temporal variability, which is due to bioclimatic, geomorphological, and land-use conditions and is highly influenced by the passage of time, such as natural or anthropogenic influences. (Dufour et al., 2019). Riverine vegetation offers a great variety of ecosystem services (Naiman et al., 2010). These areas play an essential role in the reduction, through filtering and transformation, of nonpoint sources of pollution and nutrients (Zalewski et al., 2003), being able to eliminate more than 90% of the nitrate of the underground water that flows through them (Hunter et al., 2006). In addition, they provide bank stabilisation against flooding (Carter & Anderson, 2020). They are also able of enmeshing deposition runoff from the land, promoting bank stability and therefore minimising further loss of soil to waterways (Price & Lovett, 1999). Another benefit of this vegetation is its ability to regulate the temperature of aquatic ecosystems by providing shade for them (Pusey & Arthington, 2003).

Considering all these benefits that they provide, as well as the impacts that have been previously indicated, the need to specifically regulate these areas and the natural resource arises. Specifically, different directives are established to stimulate stream restoration in Europe, particularly the Directive Floods (Directive, 2007), the Water Framework Directive (Directive, 2000), and the Habitats Directive (Council Directive, 1992). Directive 92/43/EEC considers river networks as areas of high conservation interest, so their maintenance is crucial. The Water Framework Directive aims to achieve “good ecological status” but does not include an indicator that attributes riparian areas to their relationship with the biological quality. However, advances in this sense appear in other management sectors that do not have direct competencies in these riparian areas but whose impact can be very important, as is the case of agriculture. The new CAP (common agricultural policy) also introduces the concept of “eco-schemes” to incentivize farmers to adopt measures that contribute to environmental and climate objectives (European Commission, 2021) in this respect. Such as the economic benefit to farmers who maintain and converse the riparian vegetation.

In order to achieve this, management actions must be based on accurate and updated information on the status of riparian vegetation (Council, 2002). Regional or national programmes for monitoring the health of riparian ecosystems have therefore been established in many countries (Directive, 2000). However, due to the complicated spatial layout as well as the difficult access to riparian ecosystems, field data collection increases the workload, especially when large areas are involved (Johansen et al., 2007a, b).

Remote sensing has a great potential to obtain continuous data over a broad variety of scales and resolutions, as well as for assessment and monitoring of natural resource management (Kumar et al., 2015). This technology has been recently used for studying fluvial environments, water quality, or riparian zones (Tomsett & Leyland, 2019; Topp et al., 2020). Recent advances in satellite techniques and programmes have also generated a wider availability of very high-resolution datasets that will assist in assessing better the interlinkages between key riverine vegetation characteristics (Pace et al., 2021). Currently, there are multiple open-access satellite programmes, such as the Landsat-8 (launched in 2013), Sentinel-2A (launched in 2015), and Sentinel-2B (launched in 2017) sensors, but with a non-high average resolution, but providing images of the entire surface of the Earth every few days for free (Li & Roy, 2017). However, the use of high-resolution satellite data, such as WorldView-2, would provide greater accuracy in analyses requiring higher precision, providing through its panchromatic layer a resolution of 0.5 and 2 m through the multispectral layers (Farooqi et al., 2020).

The main objective of this study is the use of high-resolution satellite imagery for the assessment and analysis of riparian ecosystems. For this purpose, the RSQI is used in multispectral satellite images provided by the WorldView-2 satellite. The data has been calibrated and validated using an index based on field data, the QBR (from its acronym in Catalan), as well as using the Land Cover and Use Information System of Spain (SIOSE) (Gobierno de España, 2016). Furthermore, the development of this methodology has a special interest to guarantee the health of these ecosystems in a study area with very different characteristics from other areas. The study area is made up of small-sized plots, smallholdings, high fragmentation in terms of ownership, and old owners, which leads to low innovation. It is, therefore, of special interest to develop, apply, and adapt this methodology in the studied area. Obtaining this index will improve the understanding and management of these areas and consequently improve the conservation, restoration, and assessment of these ecosystems.

## Materials and methods

### Study area and dataset

**Fig. 1:**
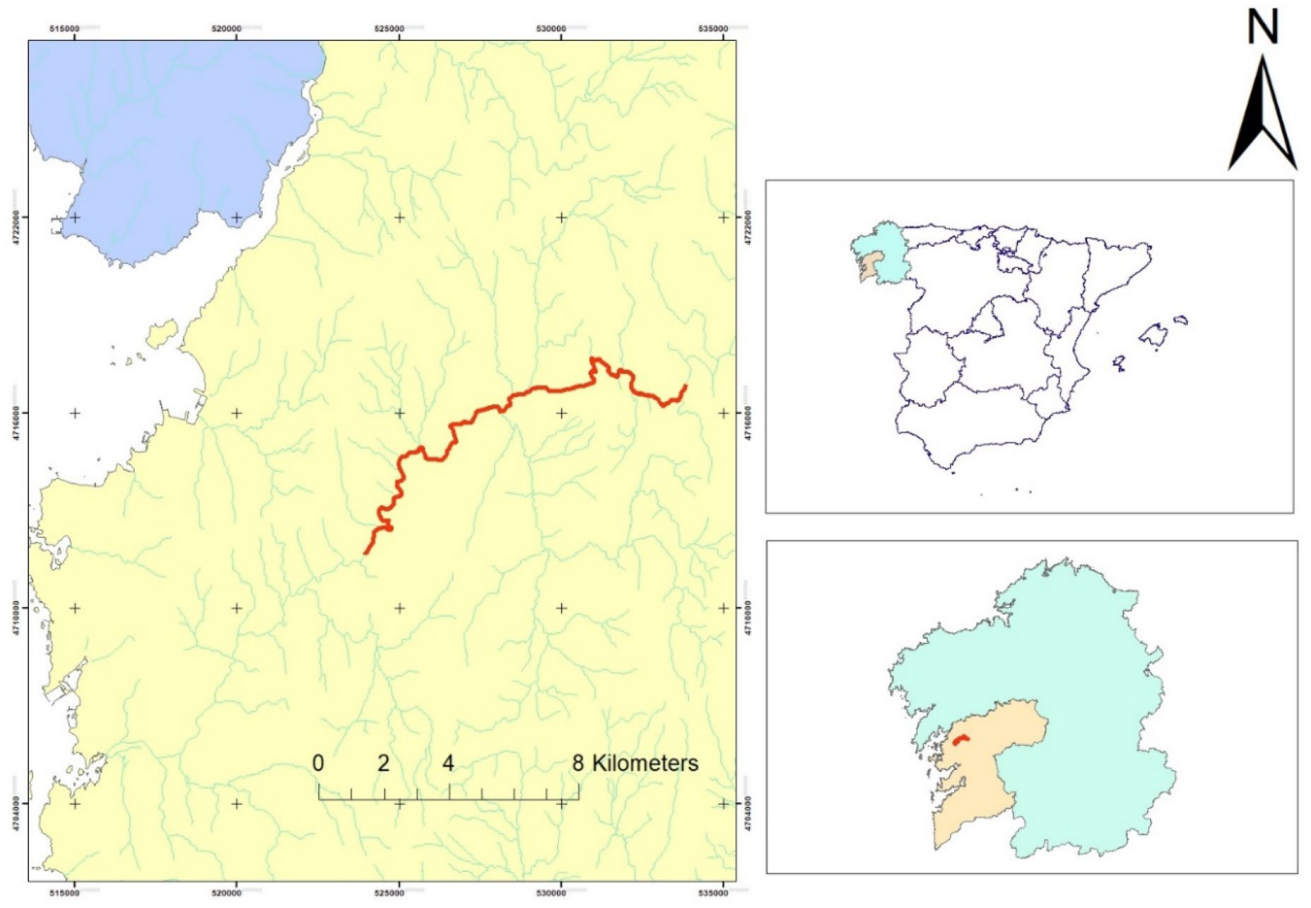
Study area, locating the Umia Basin, and the Umia River in Pontevedra (Galicia) (UTM 533823E 471635 N)

The Umia River is situated in the southwest of Galicia (northwestern Spain) (Fig. 1). The basin of this river, covering 440 km^2^, has a total length of 70 km and an average flow of 16.2 m^3^/s, and drains into the Atlantic watershed of Galicia (Álvarez et al., 2014). The region has an oceanic climate, the average annual rainfall is 1500 mm, and the average temperature is 14.8 °C (Carballeira, 1983; Cortizas & Alberti, 1999). Consequently, the peak flow period corresponds to the months of December to May, and the minimum takes place in August (de Galicia, 2008; Hilty et al., 2012). Average rainfall in the region approaches 282 Hm^3^/year (2018), temperatures vary from 7.3 °C in January to 19.5 °C in the warmest months (July–August 2018).

According to the “vegetation series map” described by Rivas-Martínez (1987), the study area belongs to the Eurosiberian region, climatological series. The study area in this work corresponds to a riverbed, so the vegetation in these specific sections belongs with the association Galician-Asturian Valeriano pyrenaicae-Alnetum glutinosa. The main riparian species identified in the study area are oak (*Quercus robur*), black alder (*Alnus glutinosa*), willow (*Salix atrocinerea*), hazel tree (Corylus avellana), elder (Sambucus nigra), and ash (*Fraxinus sp.*) among other arboreal, shrub, and herbaceous species (Appendix A). The ecological importance of these systems is known by the designation of some riparian formations as a priority habitat in Directive 92/43/EEC as alluvial forests of *A. glutinosa* and *Fraxinus excelsior* (code 91E0) (Council Directive, 1992) (Bermúdez et al., 2015; Valero et al., 2014). Land uses in the basin represent 35% of the broad-leaved forest, 24.8% of complex cultivation patterns, 15.6% of moors and heathland, 10% of coniferous forest, and 15% for other land uses (SIOSE, 2016). The Umia River Basin has a high rate of population dispersion with a total of 184 villages; 70% have 50 inhabitants, and only 2 villages have more than 500 inhabitants (Instituto Galego de Estatística (IGE), 2015).

In addition, it should be highlighted that the riparian vegetation in the study area suffers from multiple pressures. In the municipality of Caldas de Reis, the main municipality of the study area, there are around 72,000 plots categorised as rural use, with a total surface area of 6149 ha, belonging to 7500 landowners, which leads to the conclusion that the average surface area of each plot is minimum (Acuña-Alonso et al., 2022; Instituto Galego de Estatística (IGE), 2020). This is a problem specific to the community of Galicia, where 97.3% of the forest area belongs to private forests, with an average area of 0.26 ha. Furthermore, this forest area is characterised by a high fragmentation in terms of ownership, and more than two thirds of the forest land in Galicia belongs to 670,000 owners. Approximately 10,000 ha of this area corresponds to the so-called potential agricultural area, which includes pasture, grassland, fodder crops, and annual crops. On the other hand, the regulation of the public hydraulic domain (Ministerio de Obras Públicas y Urbanismo, 1986) it specifies that the owners of the easement zones (5 m from the slope) may freely sow and plant non-tree species, while for the plantations of tree species they will require authorization from the basin agency. These characteristics and regulations provide the ideal conditions for the replacement of forest land use by agricultural land use, as well as for the abandonment of the land. In addition to making it difficult to follow up and monitor these special protection areas.

### Riparian areas sampling and QBR index

Firstly, it was necessary to estimate the quality and condition of the riparian forest in order to analyse the results of the high-resolution satellite images, for which purpose the QBR (from its acronym in Catalan) has been selected. The QBR index develops a methodology that integrates biological and morphological aspects of the river and its floodplain and is used to assess and monitor riparian vegetation. This index is a simple method to evaluate riparian habitat quality. It was developed to be used in Mediterranean streams of Spain (Munné et al., 1998), and several authors have made some changes to adapt it to other geographical areas (Kazoglou et al., 2010; Sirombra & Mesa, 2012) while maintaining the basic structure and assessment procedure. In this work, the adaptation made by Valero et al. (2014, 2015) for this study area has been used. According to this index, the quality of riparian vegetation is classified by the following categories: > 95 would correspond to the “very good” category, 75–90 would be evaluated as “good”, 55–70 would correspond to “moderate”, 30–50 as “poor”, when degradation is high and < 25 “bad” when degradation is extreme.

**Fig. 2:**
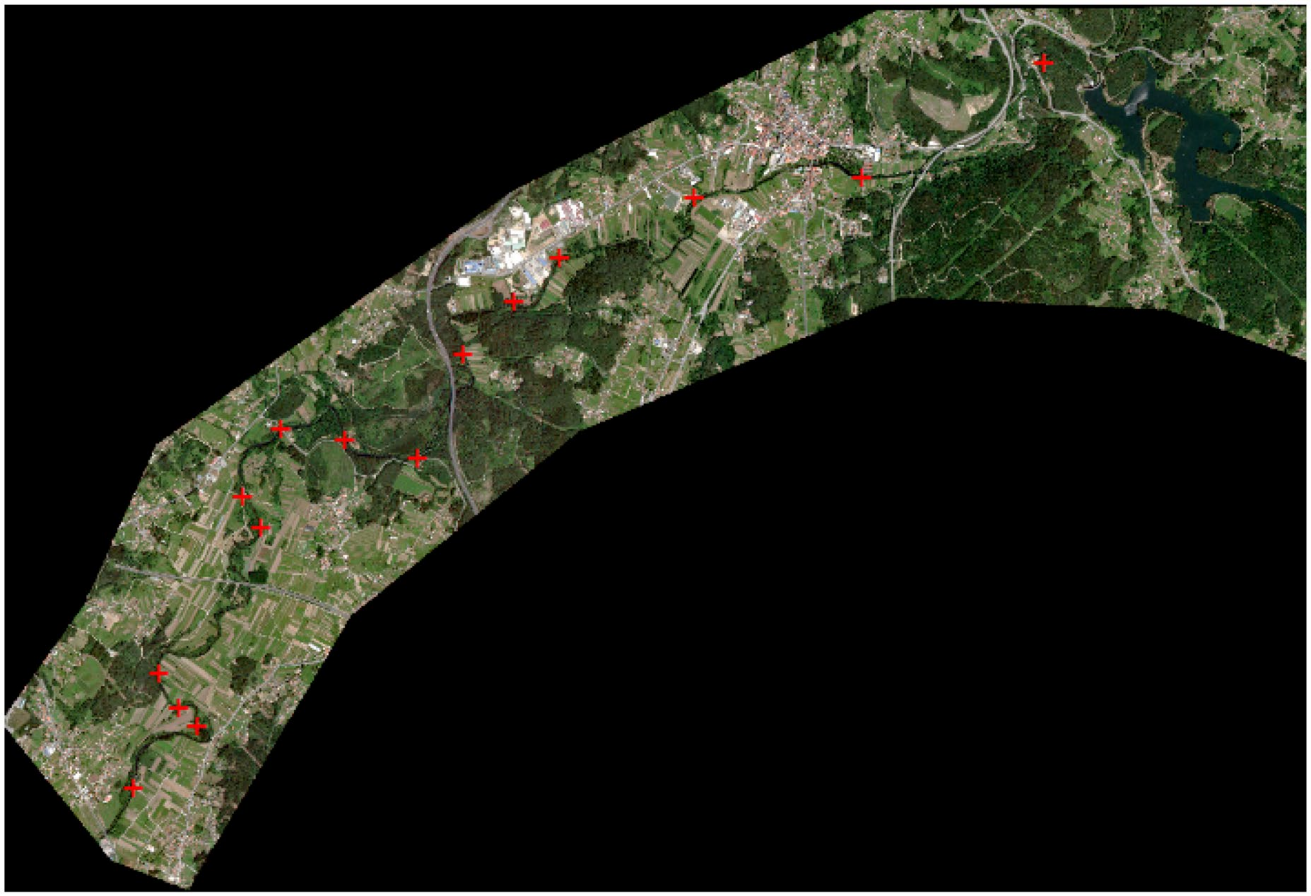
Sampling points distributed along the river

A total of 15 sampling stations of 100 m were selected along the stretch of the riparian Umia River (50 m upstream and 50 m downstream of each point) (Fig. 2). Due to the large differences between the two banks of the 15 selected sampling stations, each bank has been analysed separately, so that 30 points are evaluated. Each of the sampling stations was selected based on factors such as particular river systems, alterations by human activities, undisturbed areas, etc.; positioned (UTM coordinates) with a GPS GPSMAP 60CSx (Garmin, Olathe, KS, USA). The field period for the sampling was August 2020.

### Image acquisition and preprocessing

Images from the WorldView-2 satellite (DigitalGlobe, Inc., Westminster, CO, EE. UU.) were selected. WorldView-2 is a very high-resolution (VHR) satellite launched in October 2009 (Aguilar et al., 2016). This satellite product has a panchromatic image (PAN) at a 0.5 m spatial resolution and one multispectral image (MS) at a 2 m spatial resolution with the radiometric accuracy recorded in 16 bits. The multispectral image is composed of eight bands: coastal blue (band 1, 400–450 nm), blue (band 2, 450–510 nm), green (band 3, 510–580 nm), yellow (band 4, 585–625 nm), red (band 5, 630–690 nm), red-edge (band 6, 705–745 nm), NIR1 (band 7, 770–895 nm), and NIR2 (band 8, 860–1040 nm) (Noviello et al., 2013).

Two satellite images were obtained from this satellite, one from July 2018 and the other from May 2019, in order to analyse the spatial variation caused by seasonality. The atmospherically corrected images were processed using the PANSHARP2 algorithm (Zhang, 2002), which is integrated into ArcGIS (ESRI, 2012). Through the use of pansharpening algorithms, a high spatial resolution panchromatic (PAN) image and a multispectral (MS) image with the high spectral resolution are combined, obtaining a new image with a high resolution and realistic representation of original MS colours. Subsequently, a normalised difference vegetation index, RE-NDVI (Gitelson & Merzlyak, 1994), was calculated for both images independently using the red-edge and near-infrared bands by means of Eq. 1.

1 $$\mathrm{RE}-\mathrm{NDVI}= \frac{(\mathrm{NIR}-\mathrm{RE})}{(\mathrm{NIR}+\mathrm{RE})}$$

The equation is composed of NIR, which is defined as the reflectance in the near-infrared band (band 7), and RE, which is the reflectance in the red-edge band (band 6).

### Principal component analysis

Subsequently, a principal component analysis (PCA) was carried out for the images obtained from WorldView-2. The main matrix was formed by all the pixel values of the 8 multispectral bands. From this matrix, a second band correlation matrix was calculated, where the relationship between the bands was obtained. This process was carried out using the Rstudio software (RStudio: Integrated Development for R, 2020). The objective of the application of this methodology is to reduce and synthesise the spectral variability within the eight spectral bands of each image. Accordingly, a four-band raster file was obtained by combining the PCA and the RE-NDVI calculated for each image. This combination has been chosen since it resulted effective in other studies such as Johansen et al. (2007a, b); Novoa et al. (2018).

### Object-based image classification

Object-based image classification (OBIC) relies on the segmentation of groups of pixels to create objects and work with them at different scales using spectral, geometric, thematic, and topological information, which create a richer framework to extract geospatial information (Blaschke, 2010). The eCognition software is relying on a multi-solution segmentation algorithm to group similar pixels into objects (Novoa et al., 2018; Silva et al., 2018).

The OBIC was performed inside a buffer of 50 m around water streams, which was considered adequate as the required width of riparian strips in the study area ranges from a minimum of 3 to a maximum of 15 m. The segmentation parameters set are scale (which controls the size of the objects), form, and compactness, which controls the shape of objects and the dependence on spectral values, respectively. The selected scale parameter was 25, while the shape and compactness parameters were both 0.1. These parameters were selected on the basis of those set by Novoa et al. (2018). The image segmentation was obtained using the “multiresolution segmentation” algorithm. The next step was the classification, where a total of 2505 objects were obtained in the study area, and these were grouped into four classes: water, forest, crops, and urban areas, based on the SIOSE land use layers. The forest class includes *Eucalyptus*, *Pinus*, and deciduous species, as well as shrubs. The crops category includes annual crops and vineyards. And finally, the urban areas category includes industrial, commercial, service areas, and population centres. The “hierarchical classification” algorithm systematically analysed the classes marked in the composite raster.

The last stage consisted of validating the classification obtained and carrying out a test. For this purpose, validation polygons (200) at the 95% confidence level and 10% margin of error were randomly selected and independently evaluated on screen. Through the confusion matrix calculated, the global precision and the kappa coefficient (KC) were determined. This coefficient was used to obtain the degree of agreement between the existing truth and the projected classes.

### Riparian strip quality index (RSQI)

The RSQI determine the ecological quality of riparian strips, and it is commonly used to create ecological portraits of watershed riparian areas (Saint-Jacques & Richard, 1998). It is computed using weighted riparian land cover classes, following the methodology of Saint-Jacques and Richard (1998), as follows (Eq. 2):

2 $$\mathrm{RSQI}=\frac{\sum (\mathrm{\%}{\mathrm{LU}}_{i}x{W}_{i})}{10}$$

where %LU_i_ is the land cover area percentage inside the riverine strip and *W*_i_ corresponds to the land cover class weighting factor. It is calculated through the use surfaces of the points to be studied and the value assigned to that land use. The RSQI ranges from 17 (when the quality is at its lowest) to 100 (when the quality is at its highest). The RSQI categories have been defined in MDDEFP (2008) based on empirical evidence as shown in Table 1.

**Table 1:** Categorisation of RSQI

**RSQI categories**	**RSQI values**
Very low	< 40
Low	40–60
Moderate	60–80
High	> 80

The weighting for each of the land use was carried out following the criteria of Saint-Jacques and Richard (1998): forest (10.0), crops (1.9), bare soil (1.7), and infrastructure (1.9). This calculation was performed automatically in ArcGIS software, based on the areas of each land use at each sampling point and the value assigned to that land use according to the criteria explained adapted to Novoa et al. (2018).

### Statistical analysis

The experimental data were analysed and plotted using IBM SPSS (version 16.0; SPSS Inc, Chicago IL). Two tests were used: the Shapiro–Wilk test and homogeneity of variance using Levene’s test. The data obtained showed a normally distributed (*p* > 0.05 for both the tests) and were therefore subjected to parametric tests, where it was required. The descriptive statistics were performed for the RSQI and QBR indices calculated in this study. Finally, Pearson’s correlation was computed for both indices.

## Results and discussion

### Image preprocessing

The results obtained from the QBR index indicate that the quality of the riparian vegetation is between moderate or beginning of alteration, with a score of 56 out of 100 (Appendix A). The values obtained do not reveal any stretch of the river with a bad coefficient or extreme degradation, although the presence of multiple invasive species can be highlighted; *Acacia dealbata*, *Acacia melanoxylon*, *Arundo donax*, *Phytolacca americana*, *Cortaderia sellona*, *Robina pseudoacacia* (e.g. points 9 left and 10 left*)*. On the other hand, the presence of autochthonous species (*Alnus glutinosa*, *Salix atrocinera*, *Corylus avellana*, *Sambucus nigra*, *Fraxinus excelsior*, *Laurus nobilis*, *Quercus robur)* would improve the quality of the riparian vegetation, and consequently, the QBR index was higher in those stretches of the river where these species were found (e.g. points 1 right, 8 left, and 12 left).

**Fig. 3:**
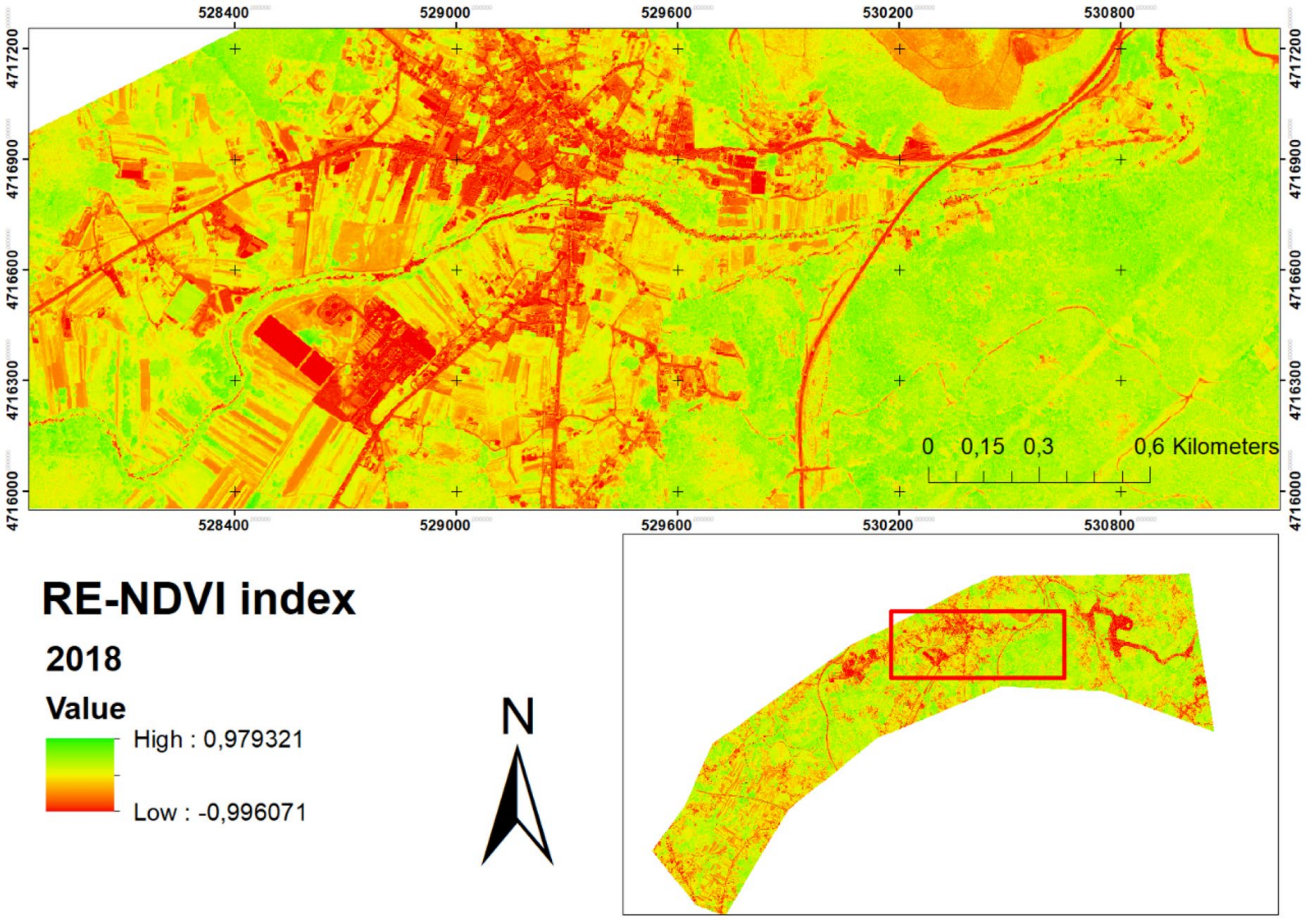
RE-NDVI vegetation index obtained for July 2018

**Fig. 4:**
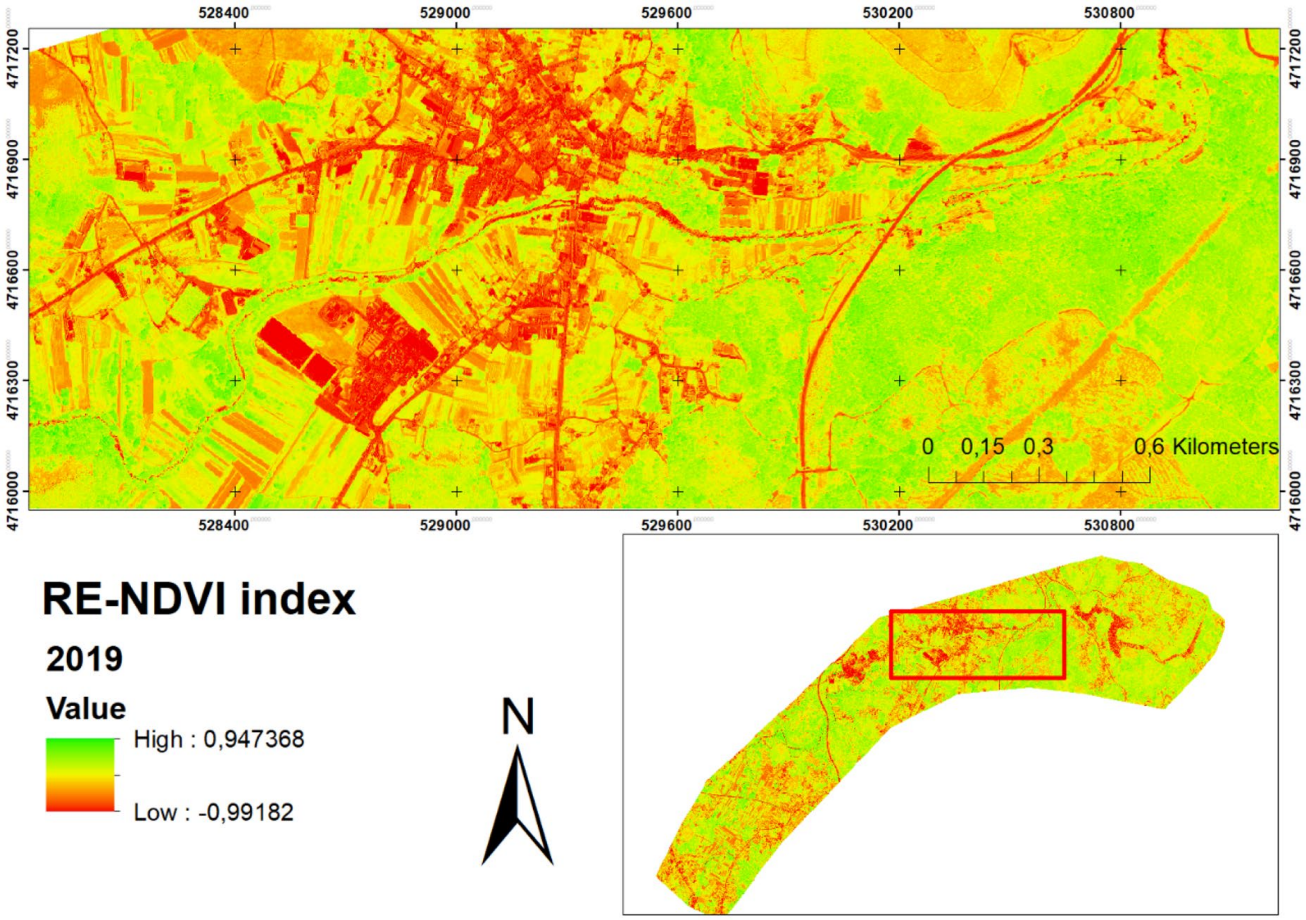
RE-NDVI vegetation index obtained for May 2019

**Fig. 5:**
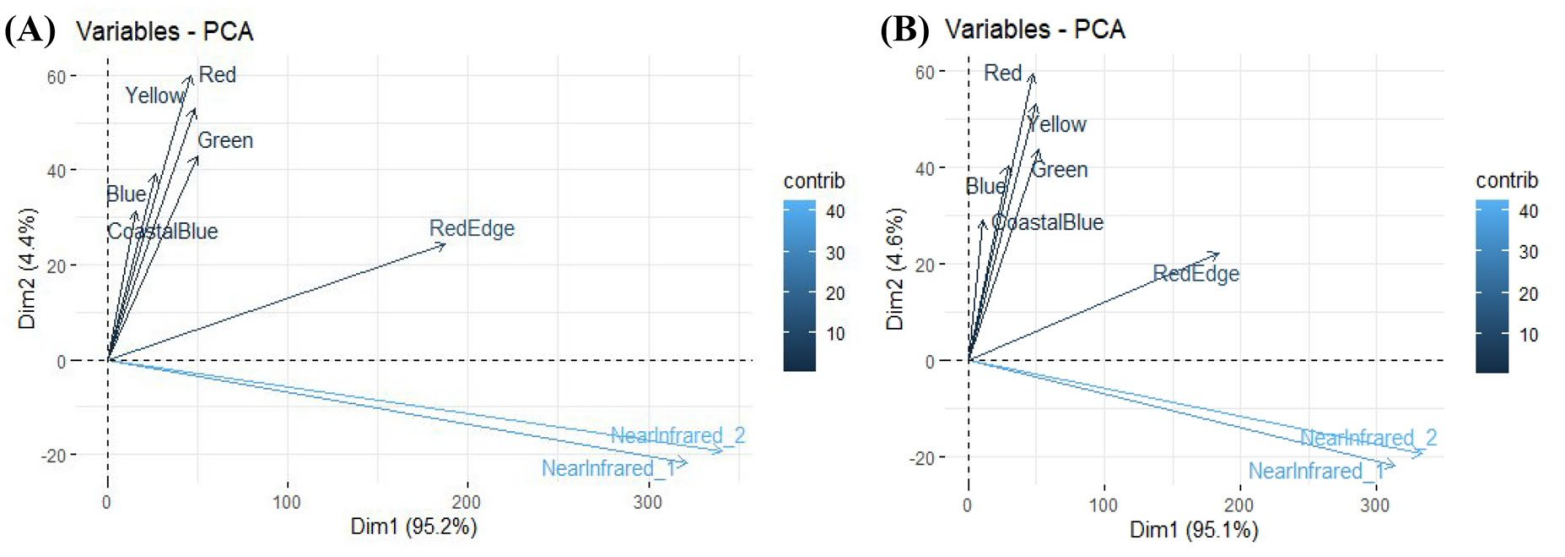
Principal component analysis; **A** image 2018; **B** image 2019

The calculation of the RE-NDVI for July 2018 (Fig. 3) and May 2019 (Fig. 4) shows slight differences in the reflectance of each season. For this reason, these images were unified as detailed in the methodology. The results show the association of primary productivity based on the RE-NDVI index, through observation and quantification using the WorldView-2 satellite. The data obtained for the RE-NDVI in 2018 provides a range between 0.07 and 0.45 and a mean value of 0.32 ± 0.06. For the spring of 2019, those values are 0.06 − 0.49 and a mean value of 0.31 ± 0.08. Al-Doski et al. (2013) concluded that sparsely vegetated NDVI values range from 0.2 to 0.4; moderately vegetated values range from 0.4 to 0.6, while densely vegetated NDVI values range from 0.6 to 1. Also, NDVI values below 0.2 represent water bodies and areas without vegetation cover. Based on these values, it can be concluded that vegetation is generally sparse in the study area. The use of this index could be used as a practical tool to illustrate vegetation changes, such as loss of photosynthetic activity, partial crown dieback, or total or partial foliage drop (Pace et al., 2021). To date, hardly any research has been carried out that includes this index for the analysis of riparian vegetation. The use of the RE-NDVI index over the traditional NDVI index is useful to better discriminate vegetation types by avoiding the high saturation levels of the latter (Mutanga et al., 2012). The RE-NDVI index would perform better than NDVI in soil and vegetation analysis, with relatively low sensitivity to vegetation cover and being negligibly affected by environmental factors (Deng et al., 2018). However, most studies analysing riparian vegetation are based only on the use of the NDVI index (Hill, 2013; Pace et al., 2021), which could decrease their accuracy. In this way, a more truthful image was obtained with the vegetation to be analysed. The results show the association of primary productivity based on the RE-NDVI index, through observation and quantification using the WorldView-2 satellite. The use of this index could be used as a practical tool to illustrate vegetation changes, such as loss of photosynthetic activity, partial crown dieback, or total or partial foliage drop (Pace et al., 2021). The use of the RE-NDVI index over the traditional NDVI index is useful to better discriminate vegetation types by avoiding the high saturation levels of the latter (Mutanga et al., 2012). The RE-NDVI index would perform better than NDVI in soil and vegetation analysis, with relatively low sensitivity to vegetation cover and being negligibly affected by environmental factors (Deng et al., 2018). However, most studies analysing riparian vegetation are based only on the use of the NDVI index (Hill, 2013; Pace et al., 2021), which could decrease their accuracy. A PCA raster was created to obtain 95.17% of the spectral variability of the original bands for the 2018 image, and 95.05% for the 2019 image (Fig. 5).

### Object-based classification

**Fig. 6:**
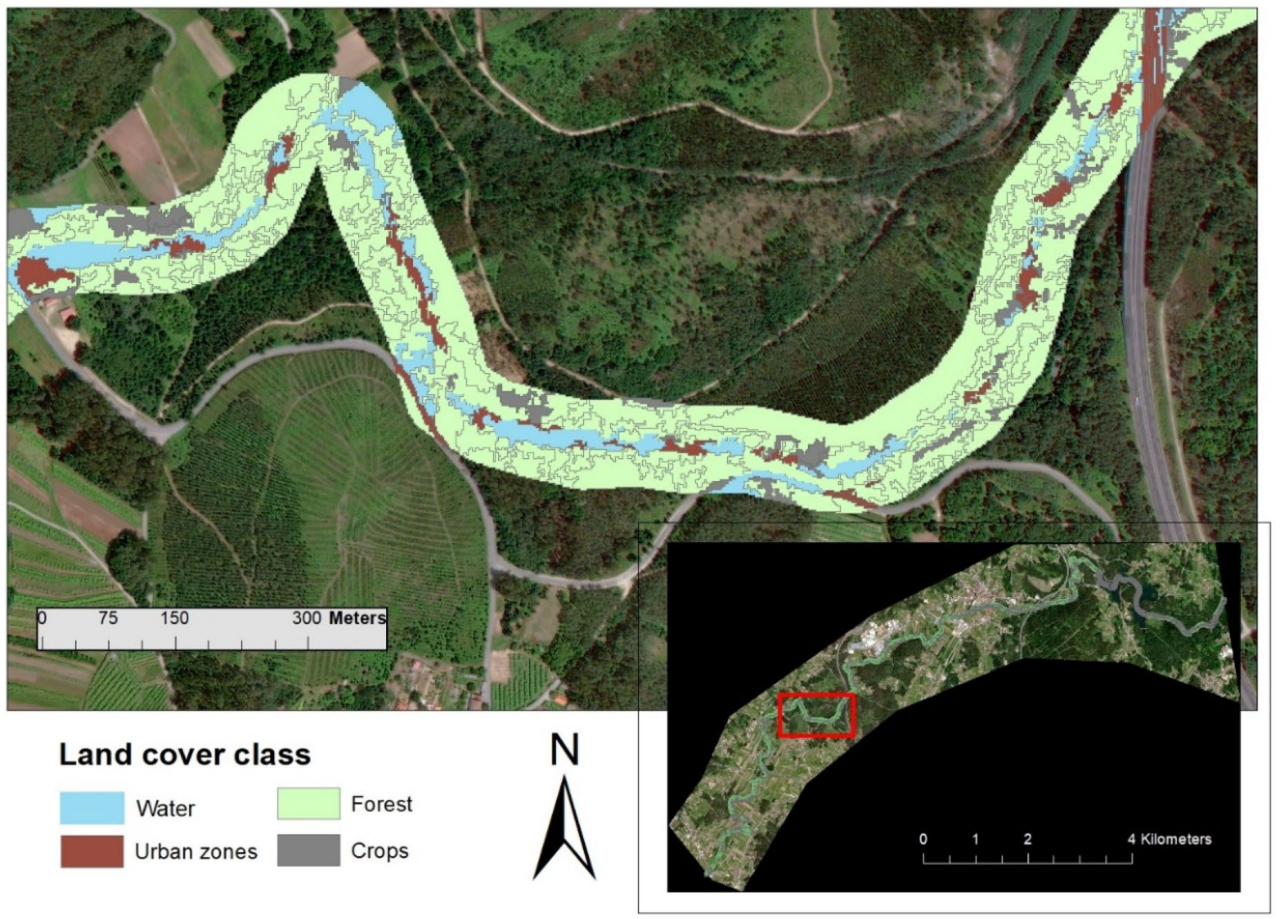
Land cover class throughout the study area following classification criterion

The OBIC performed effectively, achieving an overall of 0.88. The 50-m buffer area used as the target area to extract riparian vegetation covered 1.78 km^2^ (Table 2). Forest was the dominant class within the target area, covering 48% of the riparian zones (0.99 km^2^) (Fig. 6). The second most important land cover class extracted was water, covering 22% (0.31 km^2^). The remaining corresponds to crops (20.0%; 0.32 km^2^) and urban zones (11%; 0.19 km^2^). This distribution of land use obtained with large crops is due to the main agricultural activity in the basin with large livestock production, and the presence of poultry farms that largely dominate other livestock explorations (Álvarez et al., 2017). In general, the forest area is distributed towards the headwater of the study area; however, it is heavily degraded towards the middle due to the strong presence of urban areas. It should be noted that this forest area is deteriorated by the presence of invasive plants, as well as by the anthropogenic alteration caused by the presence of a road that crosses the area. The improvement of this forest area would reduce the risk of flooding in the area, as well as the general quality of the water (Acuña-Alonso et al., 2021). The presence of crops is found throughout the study area, the high percentage of this type of soil is due to the availability and type of plots that define the study area. These plots are small and have multiple owners, which leads to the owner wanting to take advantage of and maximise the area under cultivation. In these types of agricultural areas, most ecological problems are expected to occur, due to hydromorphological and environmental pressures, as well as land cover degradation related to human activities and agricultural practices (Henriques et al., 2015). This leads to the alteration and reduction of the forest area, as well as the riparian vegetation that acts as a green filter, reducing the presence of compounds derived from agriculture and livestock in the water (Valero et al., 2014). The improvement of this area would therefore be considered a nature-based solution (NBs) in accordance with Aikas et al. (2019).

**Table 2:** Number of objects created during the OBIC, their total percentage and areas obtained by this methodology, and the System of Land Occupation (SIOSE)

**Land cover class**	**Objects**	**%**	**Area** **(km**^**2**^**)**	**Area SIOSE** **(km**^**2**^**)**
**Forest**	546	22%	0.31	0
**Crops**	1192	48%	0.99	0.85
**Water**	499	20%	0.32	0.86
**Urban zones**	268	11%	0.19	0.16
**Total**	2505	100%	1.78	1.78

Validation statistics were calculated using 200 objects selected at random in proportion to the number of objects in each land cover class (95 forest, 44 water, 40 crops, and 21 urban zones). The forests showed the highest KC (KC > 0.89), while crops, water, and urban zones showed the lowest, with a range of 0.38 to 0.20 (Table 3). The verification of the data obtained through the mapping of images (RS QI) was compared with data taken in the field (QBR index), obtaining a value of 92% of truthfulness and a kappa coefficient of 0.88 (very good). Above study Fernandes et al. (2014), was obtained a kappa coefficient of 0.77. In this study, they also used WorldView-2 satellite imagery, in this case, to detect objects in riparian habitats. For their part, Adam et al. (2017) investigated the ability of WorldView-2 imagery to map the invasion of a plant and obtained a kappa value of 0.84.

**Table 3:** Image classification confusion matrix of the OBIC. There is the number of pixels classified in each land cover class versus those selected in the validation process (reference class)

**Land cover class**	**Reference class** **(area** **%)**			
	**(1)**	**(2)**	**(3)**	**(4)**
**(1)** **Forest**	89	3	2	1
**(2)** **Crops**	2	38	1	0
**(3)** **Water**	1	1	37	5
**(4)** **Urban zones**	0	0	1	20
**KC**	0.88			

Novoa et al. (2018) relate the low KC results to the small number of spectral bands used (RE-NDVI and PC1). Although the use of 4 bands sped up the slow process of object-based image classification, this number is insufficient for classification.

### Comparison between RSQI and QBR index

**Fig. 7:**
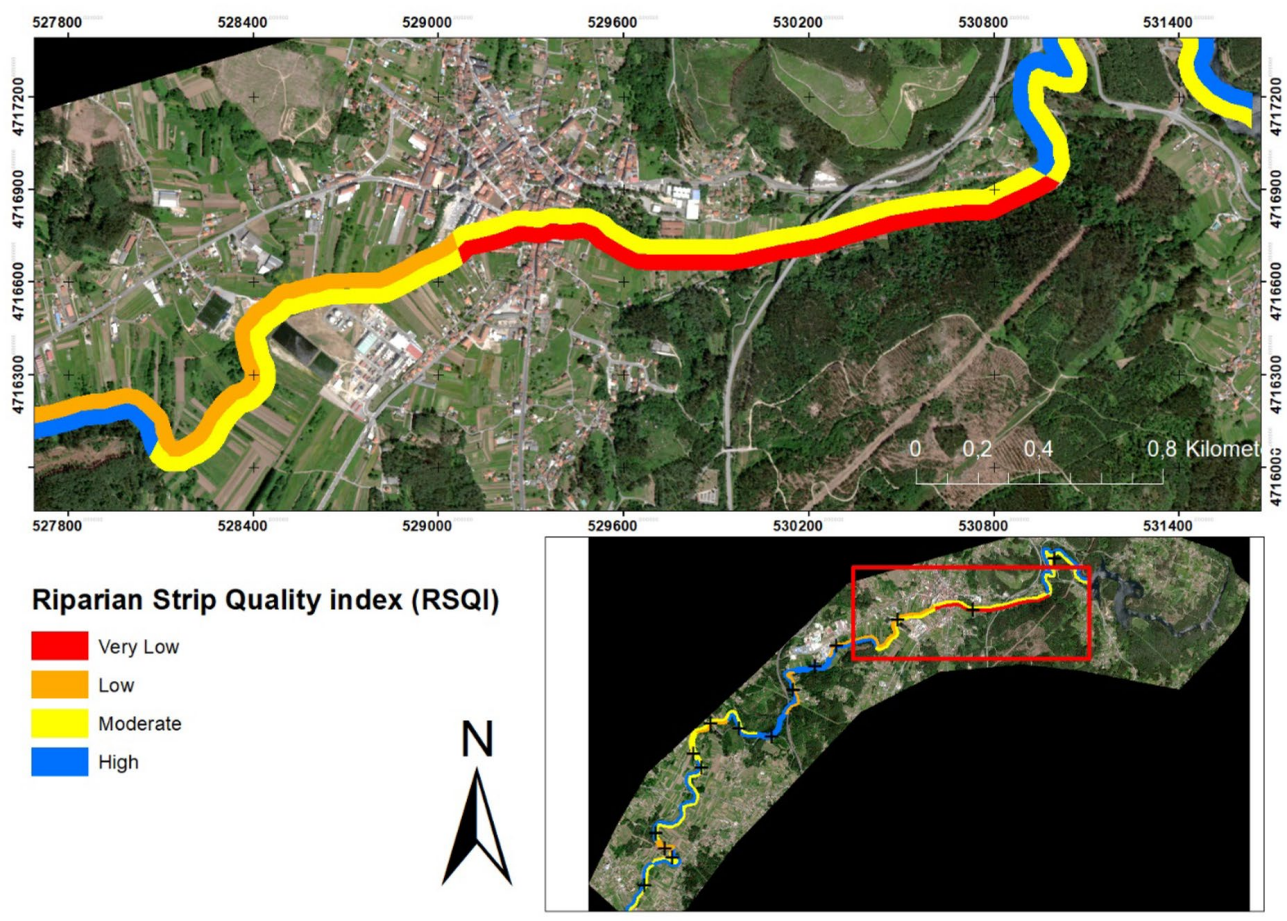
Spatial distribution of RSQI: at the catchment level (bottom right panel) and a section of the worst quality area

The average ecological quality index RSQI in the river basin was 71.57 ± 17.71, which ranks it as a watershed with moderate ecological quality (Fig. 7). According to this index, values along the river ranged from 37 being the lowest to 94 being the highest. According to the scores set by Saint-Jacques and Richard (1998), about 40.00% were of “high” quality, about 36.66% showed “fair” quality, while 20.00% were of “low” quality with visible signs of disturbance, and only 3.33% showed “very low” quality typical of extreme disturbance.

**Fig. 8:**
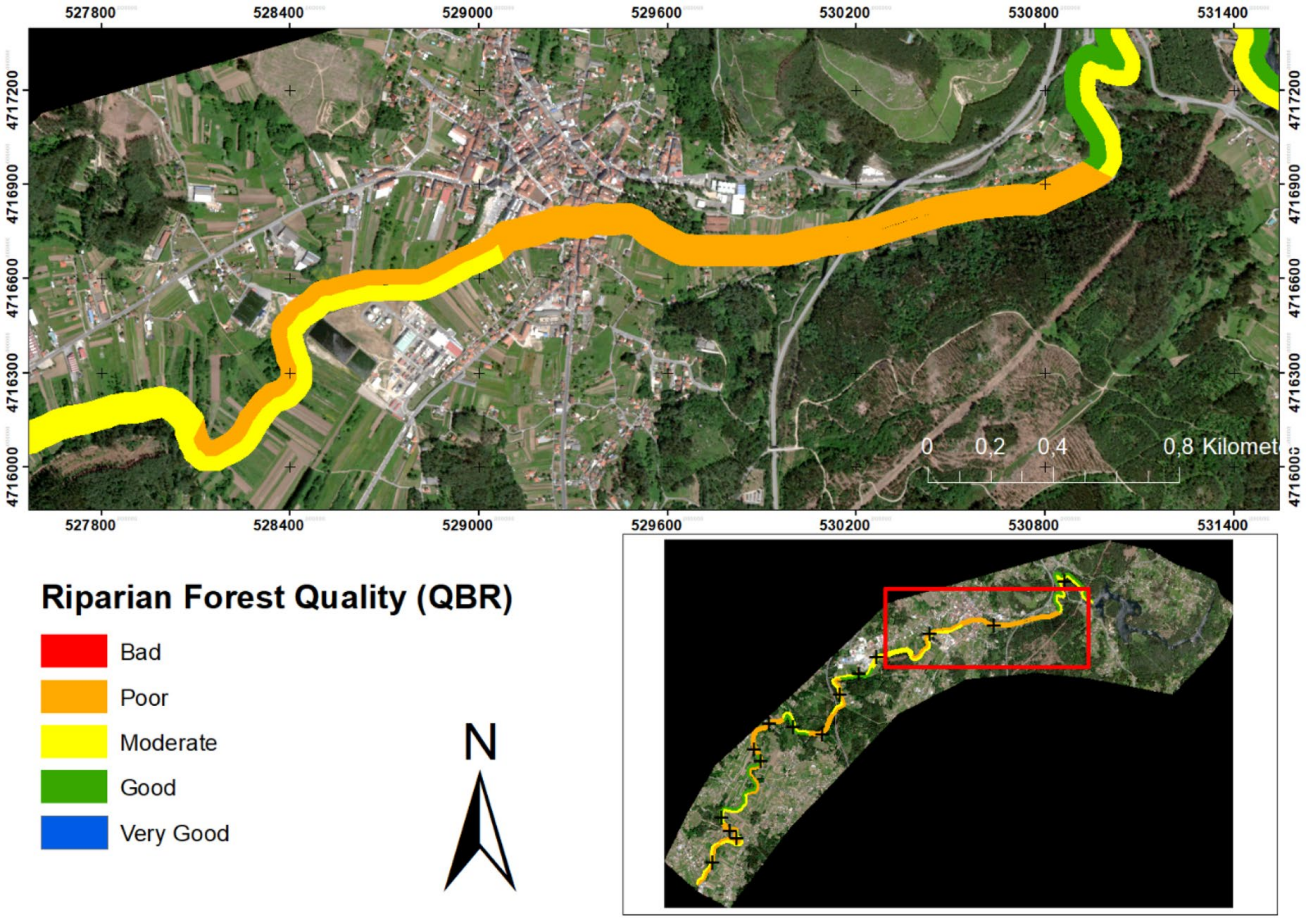
Spatial distribution of QBR: at the catchment level (bottom right panel) and a section of the worst quality area

The QBR index obtained a mean of 55.83 ± 15.03, which translates into a difference of 15.74 with the RSQI index. This difference between the indices may be due to the fact that the RSQI does not differentiate between vegetation types and does not assess the decrease in vegetation quality caused by the presence of invasive species. Or due to the presence of small anthropogenic disturbances, the vegetation cover obstructs its visualisation by means of satellite images, landfalls, or other types of small disturbances, whose resolution the satellite does not value. According to the scores set by Munné et al. (1998), about 16.66% were of “good” quality, about 50.00% showed “hair” quality, while 33.33% were of “poor” quality with visible signs of disturbance, and none showed “very poor” quality due to extreme disturbance (Fig. 8). The highest values correspond to those points with vegetation cover between 80 and 50%, in addition to presenting connectivity between the riparian forest and the adjacent forest ecosystem, and low presence of invasive plants. Meanwhile, low values correspond to low connectivity between riparian vegetation and the forest ecosystem (< 10%), low vegetation cover (< 10%), or the presence of invasive plants. In addition, in both high values (e.g. 1 right) and low values (e.g. 15 right), anthropogenic constructions are found, which only indicates the high pressure on the study area. Interestingly, no sites were in the excellent riparian quality category (QBR score ≥ 95), which would indicate that the river channel had experienced some degradation. This would highlight the importance and improvement of these ecosystems. Furthermore, Pearson’s correlation between the indices was calculated, and a moderate correlation of 0.538** (*α* = 0.01) was obtained. Higher than that obtained by Saha et al. (2020), where both indices were calculated, and they define the correlation between them as non-significant (0.462, *α* < 0.001). However, they obtained a high relationship between RSQI and NDVI (0.717, *α* < 0.000). In addition, they obtain a higher average score in the QBR index than in the RSQI. This could be due to the nature of the indices used, as the former is a qualitative index, while the latter would be quantitative, therefore more objective. This would indicate the continuous degradation of this important habitat, as well as the lack of tools to improve it. This degradation is mainly due to anthropogenic interventions in the catchment (Acuña-Alonso et al., 2021), which alter this important landscape and the ecosystem services it provides (Forman & Godron, 1981). Throughout the study area, the areas with the worst quality correspond to those with industrial areas or population centres nearby.

Following the criteria of Valero et al. (2014), where they defined different sections according to the restoration action required in the same watershed but upstream of the reservoir. This criterion was classified as follows: d: (1) conservation (QBR > 90), (2) recovery (QBR 55–90), when the riparian forest is degraded, and vegetation cover is disturbed (specific action will be needed such as removal of invasives, removal of debris, etc.), and (3) restoration (QBR < 50), in areas where the riparian forest is totally destroyed or is so unstructured that it needs to be re-established. In our study area, we would obtain that there is no conservation area, and 47% would correspond to recovery, while 53% would correspond to restoration. Considering the problems affecting our study area, it is necessary to take action to restore the riparian forest and counteract the negative effects of deterioration. Restore all the ecosystem services that these habitats can provide, e.g. by enhancing biodiversity or reducing and filtering pollution from major anthropogenic pressures such as livestock farming.

### General discussion

Remote sensing-based techniques have proven to be an efficient approach for mapping different land uses. Most studies analysing forest vegetation are based on the Sentinel-2 satellite (Krtalić et al., 2021). These are mainly based on the use of logarithms (Brovelli et al., 2020; Furuya et al., 2020) or vegetation indices (Hill, 2013; Nouri et al., 2014; Pace et al., 2021). The main advantage of these procedures is the use of low-cost images, in addition to the fact that they enable environmental monitoring and allow the adequacy of forest management decision-making. In our case, due to the resolution of these free images (between 10 and 20 m), it was decided to use very high spatial resolution images provided by the WorldView-2 satellite. Due to the fact that the riparian fringes of the study area are too narrow to be accurately assessed by satellites such as Sentinel-2. This allows for improved analysis by obtaining information from a 0.5 m panchromatic band and eight multispectral bands of 2-m spatial resolution. The free availability of these high-resolution images would be an international breakthrough and could increase the use of these images in multiple studies. In addition, these images could be used by the administration in systematic monitoring, reducing the costs of going to the field.

However, the index presented here evaluates and quantifies the quality of riparian vegetation. It provides more information than that indicated by traditional vegetation indices (e.g. NDVI) alone. In turn, it supplies more objective information than quality indices based on field forms (e.g. QBR). Providing more accurate results that could be across the entire riparian vegetation of the watershed.

The development of technologies based on remote sensing aimed at facilitating the management of natural resources provides the necessary tools to ensure comprehensive decision-making. In addition, this tool could be used to monitor restoration projects, guaranteeing the long-term follow-up of the corrective measures employed. As well, this tool could be used to monitor restoration projects, ensuring long-term monitoring of the corrective measures employed. This would make it easier to assess whether the measures used are the right ones or whether, on the contrary, new restoration measures are needed in the area. Ensuring the status of riparian vegetation can also be the basis for strategies to adapt to climate change or prevent natural disasters. It provides benefits that increase the resilience of the ecosystem to the effects of climate change, and, as a consequence, multiple ecosystem services would benefit. For example, including this methodology in the integrated management of the water cycle would benefit water quality. On the other hand, taking into account that the watershed is the fundamental unit of management, the research presented here allows improving the governance of the watershed itself through the maintenance, improvement, control, and protection of these important systems.

## Conclusions

The alteration of riparian vegetation has multiple consequences on the ecosystem services that are strongly altered, and its monitoring is essential for decision-making before reaching very high states of degradation. In the present research, RSQI has been developed using high-resolution images of the WorldView-2 satellite as a tool for monitoring riparian quality without fieldwork. The RSQI was calibrated and validated, obtaining an overall accuracy of 92% and a kappa index value of 0.88. This index, therefore, obtained a high accuracy in the classification and correct development of the index. However, in future analyses, it would be advisable to add more points as this would increase the accuracy. This index provides a tool that quantifies and identifies those areas that are more susceptible or in worse condition. The areas in best condition corresponded to stretches of vegetation with optimal cover (between 80 and 50%), good connectivity with the adjacent forest ecosystem, and little or no presence of invasive plants. While the worst scoring points had low connectivity (< 10%), low forest cover (< 10%), and a higher presence of invasive plants. The application of this index based on satellite images will facilitate the environmental governance of multiple ecosystems. Providing tools to implement best practices allows an improvement of the NBs. In this way, biodiversity will be improved, and water quality will be improved, thus guaranteeing, or improving water security.

## Supplementary Information

Below is the link to the electronic supplementary material.

Supplementary file1 (DOCX 14 KB)

## Data Availability

Data of this paper are available upon request to the corresponding author.
