# Supplementary material for: Assessment of high spatial resolution satellite imagery for monitoring riparian vegetation: riverine management in the smallholding
Source: Environ Monit Assess. 2022 Nov 7;195(1):81. doi: 10.1007/s10661-022-10667-8 (PMC9640423; doi:10.1007/s10661-022-10667-8)
Supplement: Supplementary file 1 — Supplementary file1 (DOCX 14 KB) [file 10661_2022_10667_MOESM1_ESM.docx]

| **POINT** | **QBR Index** | **RSQI Index** |
| --- | --- | --- |
| 1R | 80 | 94 |
| 1L | 70 | 67 |
| 2R | 50 | 70 |
| 2L | 45 | 37 |
| 3R | 45 | 44 |
| 3L | 55 | 77 |
| 4R | 60 | 43 |
| 4L | 70 | 84 |
| 5R | 60 | 88 |
| 5L | 75 | 92 |
| 6R | 60 | 86 |
| 6L | 50 | 47 |
| 7R | 50 | 90 |
| 7L | 40 | 87 |
| 8R | 60 | 73 |
| 8L | 80 | 85 |
| 9R | 40 | 64 |
| 9L | 30 | 45 |
| 10R | 40 | 69 |
| 10L | 35 | 79 |
| 11R | 85 | 86 |
| 11L | 45 | 75 |
| 12R | 85 | 85 |
| 12L | 60 | 68 |
| 13R | 45 | 41 |
| 13L | 40 | 46 |
| 14R | 50 | 72 |
| 14L | 65 | 91 |
| 15R | 55 | 84 |
| 15L | 50 | 78 |

APPENDIX A. Data obtained from the calculation of the QBR Index and RSQI Index.
